# Vulvar Darier-Ferrand dermatofibrosarcoma: unusual localization of a rare tumor

**DOI:** 10.11604/pamj.2019.33.46.18861

**Published:** 2019-05-22

**Authors:** Ines Zemni, Imène Sassi, Nedia Boujelbene, Sabrine Haddad, Raoudha Doghri, Riadh Chargui, Khaled Rahal

**Affiliations:** 1 Department of Surgical Oncology, Salah Azaiez Institute, University of Tunis El Manar, Tunis, Tunisia; 2 Department of Pathology, Salah Azaiez Institute, University of Tunis El Manar, Tunis, Tunisia

**Keywords:** Dermatofibrosarcoma, vulva, surgery, histopathology

## Abstract

Dermatofibrosarcoma protuberans (DFSP) is a low-to-intermediate grade sarcoma of dermal origin that rarely presents in the vulva, typically occurring on the trunk of young to middle-aged adults. Even though it has a low potential for distant metastases, it often recurs locally. Therefore, surgical excision is the treatment of choice. We report a case of DFSP with fibrosarcoma transformation involving the vulva treated by large excision with tumor free margins followed by plastic reconstruction. Our aim is to highlight this rare disease and through literature evaluate the safety and efficacy of surgical treatment. Early recognition of this rare entity whose localization and the aggressive nature of the fibrosarcomatous component will be an issue in the surgical management.

## Introduction

First described by Darier and Ferrand in 1984, Dermatofibrosarcoma protuberans (DFSP) is a low-to-intermediate grade sarcoma of dermal origin [1, 2]. The incidence of DFSP is 0.1% of all cancers and 1% of all soft tissue sarcomas that rarely presents in the vulva [1, 3]. Given the high local recurrence rate, surgical excision is the treatment of choice, and early recognition is extremely important because of the excellent prognosis following adequate excision [3].

## Patient and observation

**Figure 1:**
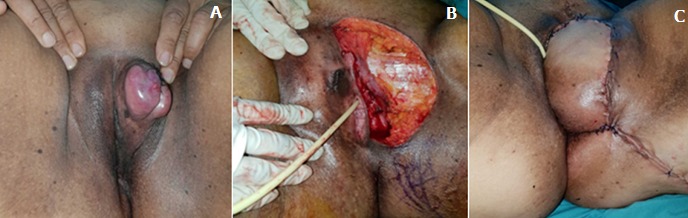
(A) a 6cm erythematous nodular tumor on the left major labia of the vulva; (B,C) wide excision with margins of 5cm and reconstruction by a lotus petal flap

**Figure 2:**
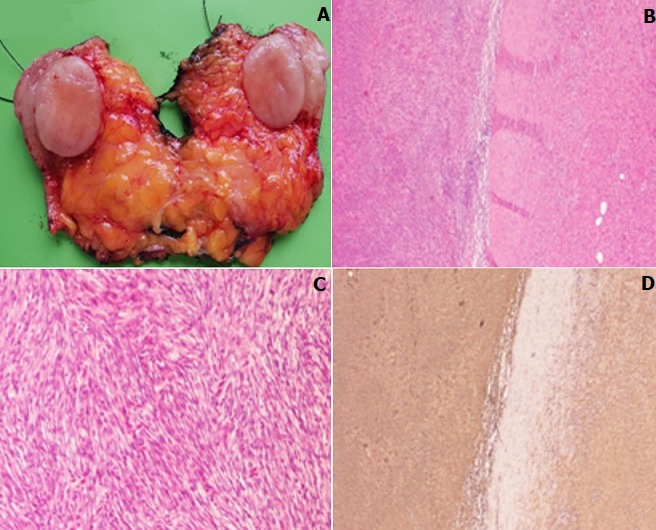
(A) cut section: well delineated and pearl-white tumor; (B) DFSP associated with fibrosarcomatous transformation: abrupt transition of typical DFSP (right) to a fascicular proliferation of spindle cells (left) (HE ×x 40); (C) with increased cytologic atypia and mitotic activity in fibrosarcomatous areas (HE x 200); (D) and diffuse CD34 positivity in both areas (IHCx40)

**Figure 3:**
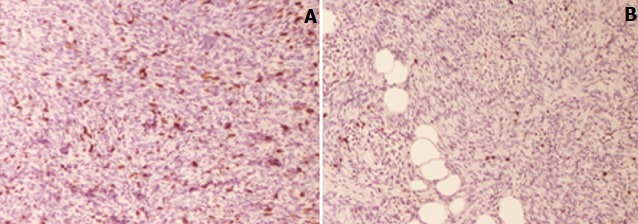
(A) Ki-67 staining: IHC, x 200: marked increase in cell proliferation in fibrosarcomatous areas; (B) IHC, x 200: lower Ki67 staining in conventional DFSP

A 47-year-old woman presented with a growing mass in the groin area. She complained of slow but progressive growth for the past 4 months. The patient had no medical or surgical history. She mentioned that the cutaneous lesion had started as a small plaque on the vulva, which was initially ignored then treated with traditional medicine. Given the continued growth of the tumor and the pain associated, the patient consulted our department. Physical examination revealed a 6cm firm erythematous nodular tumor of elastic consistency on the left major labia of the vulva (Figure 1A). After an excisional biopsy of the vulvar lesion, histological analysis showed a cellular neoplasm composed of plump spindle cells arranged in a storiform pattern, scattered pleomorphic cells. CD34 immunohistochemical study revealed diffuse reactivity. The tumor was radically excised with free margins of 5cm reconstruction by a lotus petal flap was performed by the plastic surgeons (Figure 1B,C). On sectioning, the tumor was apparently well delineated, pearl-white in color and of firm consistency, measuring 50 x 40 x 35mm (Figure 2A). Histopathologic examination showed a dermal-based neoplastic proliferation with deep extension into hypodermis and fascial tissue. The neoplastic proliferation was predominantly arranged in short intersecting fascicles with abrupt transition in depth with a herringbone architectural pattern of growth and increased cellularity (Figure 2B). In these deep areas, increased cytologic atypia and mitotic activity were noted (Figure 2C). Immunohistochemical stains (Figure 2D) revealed diffuse CD34 positivity. Tumor cells show higher nuclear positivity to Ki67 in hypercellular areas than in DFSP typical areas (Figure 3A,B). Based on the histopathologic and immunohistochemical findings, a diagnosis of dermatofibrosarcoma protuberans with fibrosarcomatous transformation (FS-DFSP) was made, having tumor-free resection margins. The patient consulted a month later with a healed incision and good esthetic state. Regular evaluations are being performed for local recurrence and distant metastasis.

## Discussion

Dermatofibrosarcoma protuberans (DFSP), previously considered in the World Health Organization classification of tumors of soft tissue [4] as a neoplasm of fibrohistiocytic differentiation, is now listed as a tumor of fibroblastic/myofibroblastic differentiation in the 2013 edition of the classification [5]. It is a superficial low-grade sarcoma of intermediate malignancy that typically arises on the trunk and proximal extremities. Even though it has a low potential for distant metastases [6], it has a high frequency for local recurrence due to diffuse infiltration of the dermis and subcutis [5]. DFSP of the vulva is extremely rare and mostly affects women in the fourth or fifth decades of life. The labia majora was most commonly affected. Patients generally presented with a 4.2cm (average) firm, asymptomatic mass. Lesions presented with erythema, hyperpigmentation, ulceration, or even of orange peel skin appearance. Patients reported slow to minimal change in size (ranging from 3 months to 10 years) [7]. As with all solid tumors, clinical suspicion is confirmed by biopsy. Histopathogically, DFSP shows a distinct “storiform” or “cartwheel” arrangement of uniform appearing fibroblasts. Immunohistochemical staining demonstrates strong positivity for CD34 (sensitivity 84-100%) and vimentin [2, 8]. DFSP can rarely present as a more aggressive fibrosarcomatous variant which occurs in around 10-15% of cases. Areas of fibrosarcoma are characterized by increased cellularity and loss of CD34 imunopositivity. Patients with this more aggressive variant tend to be more prone both to local recurrence and distant metastasis. Currently, only around 150 cases of transformed DFSP have been reported with distant metastasis in 13% [9] The initial treatment of choice for DFSP is surgery. It must be extensive as to remove the tumor completely, given its proclivity for irregular and often deep subclinical extensions. Re-resection is recommended if the initial surgery did not achieve clear margins [10].

The surgical approach to DFSP must be meticulously planned. Size, location of the tumor and fibrosarcomatous transformation, as well as cosmetic issues, will dictate the most appropriate surgical procedure. Mohs or modified Mohs surgery and traditional wide excision, typically with 2 to 4cm margins to investing fascia that is subsequently verified to be clear by traditional pathologic examination, are all methods to achieve complete histological assessment [11]. A large retrospective series of 204 patients with DFSP showed a very low local recurrence rate (1%) using wide excision with a standardized surgical approach, underscoring the importance of meticulous pathologic margin evaluation with any surgical technique [11]. A systematic review found a lower rate of recurrence with Mohs surgery compared to wide local excision [12]. Radiation has occasionally been used as a primary therapeutic modality for DFSP, but it is more commonly used as adjuvant therapy after surgery [13]. Postoperative radiation therapy is a preferred option for positive surgical margins if further resection is not feasible. If a negative margin was achieved, no adjuvant treatment is necessary. No matter which treatment option is chosen for DFSP, long-term follow up is necessary. NCCN 2018 recommends a physical exam with a focus on primary site every 6-12 months with patient education about regular self-exam. As for DFSP-FS it is recommended that the patient undergoes a physical exam every 3 to 6 months for 2 to 3 years then annually [14]. Since recurrence rates are high, some studies have suggested that follow up should include magnetic resonance imaging (MRI) to monitor for any sign of recurrence closely [15]. Some studies also suggest that patients should receive routine chest x-rays because multiple local recurrences as well as the fibrosarcomatous transformation can increase the risk for lung metastases [14, 15].

## Conclusion

DFSP is a rare occurrence, especially in the vulva. The role of immunohistochemistry with CD34 is imperative in establishing the diagnosis. Surgical excision with free margins is the treatment of choice given that the entire prognosis depends on it. The rate of local recurrence is high, but rarely are metastatic lesions present thus a frequent clinical follow-up is imperative in this rare case to detect and diagnose any recurrence of the tumor, especially for cases with a fibrosarcomatous transformation.

## Competing interests

The authors declare no competing interests.
